# Mutation in the distal NPxY motif of LRP1 alleviates dietary cholesterol-induced dyslipidemia and tissue inflammation

**DOI:** 10.1194/jlr.RA120001141

**Published:** 2020-12-15

**Authors:** Anja Jaeschke, April Haller, James G. Cash, Christopher Nam, Emily Igel, Anton J.M. Roebroek, David Y. Hui

**Affiliations:** 1 Department of Pathology and Laboratory Medicine, Metabolic Diseases Research Center, University of Cincinnati College of Medicine, Cincinnati, OH, USA; 2 Laboratory for Experimental Mouse Genetics, Center for Human Genetics, KU Leuven, Leuven, Belgium

**Keywords:** lipoproteins/receptors, cholesterol metabolism, metabolic studies, NAFLD, NASH, inflammation, LRP1, insulin resistance, ABCA1, ATP binding cassette subfamily A member 1, AKT, protein kinase B, ARC, activity regulated cytoskeleton associated protein, CD, cluster of differentiation, CCL, C-C motif chemokine ligand, CLS, crown-like structures, EMR1, EGF-like module-containing mucin-like hormone receptor-like 1, also known as F4/80 gene, Foxo1, forkhead box O1, GSK3β, glycogen synthase kinase 3 beta, HF, high-fat, HFHC, high-fat high cholesterol, IL-1β, interleukin 1β, LF, low-fat, LRP1, LDL receptor-related protein-1, LXR, liver X receptor, MCP1, monocyte chemoattractant protein-1, MIP1α, macrophage inflammatory protein-1α, PCSK9, proprotein convertase subtilisin/kexin type 9, PSD-95, post-synaptic density protein 95, TGFβ, transforming growth factor beta, TNFα, tumor necrosis factor α

## Abstract

The impairment of LDL receptor-related protein-1 (LRP1) in numerous cell types is associated with obesity, diabetes, and fatty liver disease. Here, we compared the metabolic phenotype of C57BL/6J wild-type and LRP1 knock-in mice carrying an inactivating mutation in the distal NPxY motif after feeding a low-fat diet or high-fat (HF) diet with cholesterol supplementation (HFHC) or HF diet without cholesterol supplementation. In response to HF feeding, both groups developed hyperglycemia, hyperinsulinemia, hyperlipidemia, increased adiposity, and adipose tissue inflammation and liver steatosis. However, LRP1 NPxY mutation prevents HFHC diet-induced hypercholesterolemia, reduces adipose tissue and brain inflammation, and limits liver progression to steatohepatitis. Nevertheless, this mutation does not protect against HFHC diet-induced insulin resistance. The selective metabolic improvement observed in HFHC diet-fed LRP1 NPxY mutant mice is due to an apparent increase of hepatic LDL receptor levels, leading to an elevated rate of plasma lipoprotein clearance and lower hepatic cholesterol levels. The unique metabolic phenotypes displayed by LRP1 NPxY mutant mice indicate an LRP1-cholesterol axis in modulating tissue inflammation. The LRP1 NPxY mutant mouse phenotype differs from phenotypes observed in mice with tissue-specific LRP1 inactivation, thus highlighting the importance of an integrative approach to evaluate how global LRP1 dysfunction contributes to metabolic disease development.

The LDL receptor-related protein-1 (LRP1) was identified initially as the apoE receptor in the liver that acts in parallel with the LDL receptor for plasma clearance of chylomicron and VLDL remnant lipoproteins (reviewed in [1]). Since its original discovery, LRP1 was also found to be highly expressed in numerous cell types, interacts with a wide spectrum of macromolecular substrates, and regulates cell functions in a cell type- and context-dependent manner. The importance of these LRP1 functions in health maintenance, and disease protection is best illustrated by numerous studies showing that *LRP1* gene polymorphisms are associated with a wide spectrum of diseases. For example, data from the Genetics of Lipid Lowering Drugs and Diet Network revealed a highly significant relationship between *LRP1* polymorphism and body mass index (2). Genome-wide screening of over 100,000 individuals as well as two prospective cohort studies have also identified an association between *LRP1* polymorphism with elevated triglyceride and reduced HDL levels that predisposes patients to type 2 diabetes manifestation (3, 4). In fact, a recent survey revealed that *LRP1* polymorphism is the top gene variation among 1,000 candidate gene variations in influencing fasting insulin and C-peptide levels and insulin resistance in patients with metabolic syndrome (5). These large-scale population studies suggested that LRP1 dysfunction may contribute directly to metabolic disease advancement. Another study showed that low LRP1 levels are also associated with poor prognosis of hepatocellular carcinoma (6), a pathological condition that is often associated with liver cirrhosis caused by hepatitis virus, alcoholic fatty liver, and non-alcoholic fatty liver (7). Interestingly, high dietary cholesterol intake is a major risk factor for liver cirrhosis and cancer (8). Hence, the relationship between LRP1 levels and hepatocellular carcinoma may be related to aberrant lipid metabolism in the liver.

The underlying mechanism by which LRP1 dysfunction promotes obesity, diabetes, and fatty liver disease is not completely understood. Most of the information regarding how LRP1 dysfunction may increase metabolic disease risk is inferred from in vitro cell culture experiments or in vivo studies with conditional knockout mice with tissue-specific LRP1 deficiency. Studies regarding the role of LRP1 expressed in hepatocytes have documented its importance for uptake of chylomicron and VLDL remnants (9), HDL secretion, and apoA-I lipidation (10). Expression of LRP1 in hepatocytes is also required to limit fat-induced hepatic steatosis, lipotoxicity, insulin resistance, and steatohepatitis (11, 12, 13). In adipose tissues, LRP1 has been shown to be important for preadipocyte differentiation to mature adipocytes (14, 15) and for appropriate lipid storage in mature adipocytes (14, 15, 16). The inactivation of LRP1 in mature white and brown adipocytes reduces diet-induced obesity and insulin resistance by redistributing lipid nutrients to the muscle to support thermogenesis (16), but the lack of LRP1 in mature adipocytes also increases inflammation and accelerates atherosclerosis in hyperlipidemic mice with normal LRP1 expression in the vessel wall (17). Unfortunately, embryonic lethality of mice with whole-body *Lrp1* gene inactivation (18) has precluded studies to ascertain the impact of LRP1 dysfunction at the organismal level on cardiometabolic disease risk. This study took advantage of the availability of LRP1 knock-in mice carrying an inactivating mutation in the distal NPxY motif to examine the consequence of global LRP1 dysfunction in diet-induced metabolic diseases.

## Materials and Methods

### Animals and diets

LRP1 NPxY mutant mice carrying the NPVYATL→AAVAATL mutation were generated by recombinase-mediated cassette exchange (19). The animals were backcrossed to C57BL/6J strain (Jackson Laboratories) prior to experiments. Both LRP1 distal NPxY mutant mice and C57BL/6J mice used for experiments were obtained from homozygous crosses and were housed in a facility accredited by the American Association for Laboratory Animal care. At 10 weeks of age, male mice were fed a low-fat (LF) rodent diet (LM485; Harlan-Teklad, Madison, WI) or high-fat (HF) Western-type diet without cholesterol supplementation (D12331, Research Diets, New Brunswick, NJ) or HF diet with cholesterol supplementation (HFHC) (D12108C, Research Diets) for 16 weeks as described (13). Macronutrient composition of the diets as reported by the supplier is shown in supplemental Table S1. All procedures and animal care techniques were approved by the Institutional Animal Care and Use Committee of the University of Cincinnati.

### Body composition and plasma chemistry

Body fat mass was measured in conscious mice using ^1^H magnetic resonance spectroscopy (EchoMRI-100, Echo-medical Systems) as described (16). Plasma was prepared from blood samples obtained from mice after an overnight fast. Glucose levels were measured with an Accu-Chek glucometer (Roche Applied Sciences, Indianapolis, IN), and insulin levels were determined by the UltraSensitive Rat Insulin ELISA kit (Crystal Chem, Chicago, IL). Homeostatic model assessment of insulin resistance (HOMA-IR) was calculated from fasting glucose and insulin levels using the formula [glucose (mg/dL) × insulin (ng/mL)]/22.5. Plasma cholesterol and triglyceride levels were measured using the Cholesterol Assay Kits and Infinity Triglyceride Assay Kits (Thermo Fisher Scientific), respectively. Lipoproteins in 200 μL of plasma pooled from 4–5 mice in each group were separated by fast-performance liquid chromatography (FPLC) using two Superose 6 HR 10/30 columns connected in series. Fractions of 0.5 mL were collected for cholesterol determination. The elution of the different classes of lipoproteins from the FPLC column was determined by comparison with standards. Plasma leptin, tumor necrosis factor α (TNFα), and interleukin 6 (IL-6) levels were measured by ELISA kits from R&D Systems.

### Insulin signaling

Mice were fasted overnight and treated with insulin (1 U/kg body weight) or saline by intraperitoneal injection. The epididymal fat pads were isolated after 30 min and homogenized in ice-cold radioimmune precipitation assay buffer (Thermo Fisher) containing protease and phosphatase inhibitors for Western blot analysis. For in vitro experiments, primary hepatocytes were isolated by collagenase perfusion as described (12). The hepatocytes were allowed to attach to tissue culture dishes for at least 6 h in William's E medium containing 10% fetal bovine serum, followed by overnight incubation in serum-free William's medium prior to insulin stimulation. The cells were harvested in ice-cold radioimmune precipitation assay buffer containing protease and phosphatase inhibitors for Western blot analysis.

### Cell surface protein analysis

Proteins located at the hepatocyte cell surface were analyzed by biotinylation as described previously (10). Briefly, the hepatocytes were washed with ice-cold PBS and incubated with 0.5 mg/ml sulfo-NHS-SS-biotin (Pierce) in PBS for 30 min at 4°C. After quenching with 50 mM Tris-HCl, pH 7.6, cells were harvested in ice-cold radioimmune precipitation assay buffer containing proteases and phosphatase inhibitors. Equal protein concentrations were incubated with streptavidin-agarose (Pierce) for 1 h at 4°C, washed exhaustively with PBS, and protein-biotin complexes were eluted from the streptavidin beads with 4× Tris-glycine gel loading buffer containing 10% β-mercaptoethanol at 37°C for 30 min. The eluted proteins were analyzed by Western blot analysis.

### Glucose tolerance test

Mice were injected intraperitoneally with glucose (2 g/kg body weight) after an overnight fast. Blood glucose levels were measured over a 2-h period with an Accu-Check glucometer.

### Hepatic lipid composition

Livers were homogenized in buffer containing 50 mM Tris-Cl, pH 7.4, 150 mM NaCl, and 5 mM EDTA and then extracted with petroleum ether. Triglycerides were measured using the Infinity Triglyceride Assay Kit as described above. Free and total cholesterol were measured using the Amplex Red Cholesterol Assay Kit (Thermo Fisher Scientific) according to the manufacturer's instructions.

### Tissue section analysis

Histology was performed using tissues fixed in 10% formalin, dehydrated, and embedded in paraffin. Three 5-μm sections from each mouse were analyzed for adipocyte size after staining with hematoxylin and eosin. For quantification of crown-like structures (CLSs), paraffin sections were stained with antibodies to F4/80 (Abcam), treated with a biotinylated secondary antibody followed by incubation with avidin-biotin complex (Vectastain Elite ABC; Vector Laboratories), and then visualized with ImmPACT DAB peroxidase substrate. Slides were counterstained with hematoxylin. Images of three representative fields per slide were taken in a blinded manner. F4/80-positive CLS were counted, and the total number of CLS compared with the total adipocyte number was used to quantify adipose tissue macrophage content as described (20).

### Quantitative real-time PCR

RNA was prepared using TRIzol reagent (Invitrogen) according to the manufacturer's instructions. RNA was reverse transcribed using the qScript cDNA synthesis kit from QuantaBio (QuantaBio, Beverley, MA). Quantitative real-time PCR was performed on a StepOnePlus Fast Thermocycler using Fast SYBR Green Master Mix (Applied Biosystems, Carlsbad, CA) with primer sequences as shown in supplemental Table S2. Cyclophilin expression levels were used as controls.

### Western blot analysis

Tissue homogenates or cell culture lysates for protein analysis were prepared in ice-cold radioimmune precipitation assay buffer containing protease and phosphatase inhibitors. In selected experiments, liver membranes were prepared as described previously for LDL receptor detection (21). The samples were resolved by SDS-polyacrylamide gel electrophoresis and transferred to polyvinylidene difluoride membranes (Bio-Rad). The membranes were incubated with primary antibodies (supplemental Table S3), washed and then incubated with horseradish peroxidase-conjugated secondary antibodies, and visualized by chemiluminescence (Pierce). Quantifications were performed using NIH Image J software.

### Flow cytometry

Liver was minced into small pieces and digested for 60 min at 37°C in dissociation buffer (RPMI with 15% FBS, 0.1 mg/mL DNase-I, 0.2 mg/mL collagenase P). The cell suspension was filtered through a 100-μm strainer, washed with PBS, and then resuspended in flow cytometry buffer. Cells were stained with CD45 antibodies to identify nonparenchymal cells and CD68, CD11b, and F4/80 antibodies to identify the various immune cells. Flow cytometry analysis was performed on a Guava EasyCyte 8HT System and using Guava InCyte software (Millipore, Hayward, CA).

### VLDL secretion and clearance

To assess VLDL secretion, mice were fasted overnight and then received an intraperitoneal injection of 10% Poloxamer (1 g/kg body weight) to block lipolysis. Plasma was collected by tail bleeding hourly for 3 h for triglyceride measurements. To determine plasma lipoprotein clearance, artificial lipoproteins were prepared by diluting 20% Intralipid (Sigma Aldrich) to 5% concentration with sterile saline and then sonicated in the presence of 40 μCi [^3^H]cholesteryl oleoyl ether as described (22). The radiolabeled lipid mixture (2 × 10^6^ cpm) was injected intravenously into overnight fasted mice, and blood was collected from the tail vein at 4, 10, 15, 30, 45, and 60 min to determine plasma clearance rate. Data are presented as the percentage of the radioactivity at the 4-min point after injection.

### Statistical analysis

Statistical analysis was performed using SigmaPlot, version 14.0, software (SysStat Software, San Jose, CA). All data were expressed as mean ± SE. Normality was examined using the Shapiro-Wilk test. Data with equal variance based on Levene's analysis were evaluated by Student's *t*-test or analyzed by one-way ANOVA with the Student-Newman-Keuls test for multiple group comparisons. Differences at *P* < 0.05 were considered statistically significant.

## Results

### LRP1 distal NPxY motif mutation reduces dietary cholesterol-induced adiposity and adipose inflammation

**Fig. 1:**
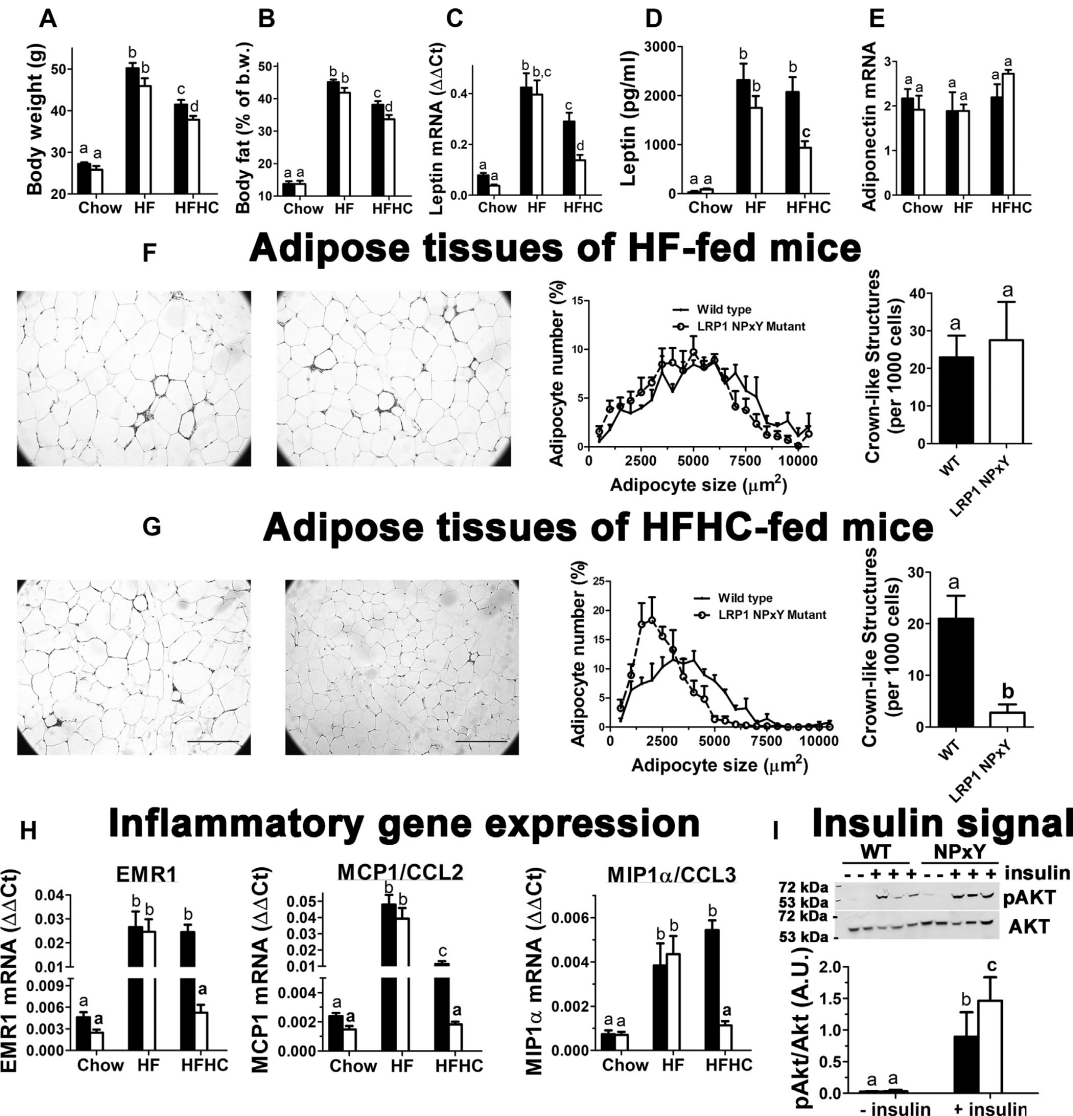
LRP1 distal NPxY motif mutation reduces dietary cholesterol-induced adiposity and adipose inflammation. A: Body weight and (B) adiposity measured as % body fat of wild-type (WT, filled bars) and LRP1 NPxY mutant mice (open bars) after feeding a low-fat chow, HF, or HFHC diet for 16 weeks (11–18 mice per group). C: Leptin mRNA levels in epididymal adipose tissue. D: Leptin protein levels in plasma. E: Adiponectin mRNA levels in epididymal adipose tissue. F: Representative histological images of HF diet-fed WT and mutant mice (left panel), quantification of adipocyte size (middle panel), and crown-like structures (right panel) from four mice per genotype. G: Representative histological images of HFHC diet-fed WT and mutant mice (left panel), quantification of adipocyte size (middle panel), and crown-like structures (right panel) from four mice per genotype. Scale bar = 100 μm. H: Expression levels of EMR1, MCP1/CCL2, and MIP1α/CCL3. Data represent mean ± SEM from eight mice per genotype. I: AKT and AKT phosphorylation in adipose tissues of HFHC-fed mice after insulin injection. Differences between groups in each experiment were evaluated by one-way ANOVA with the Student-Newman-Keuls test for multiple group comparisons. Bars with different letters denote differences at *P* < 0.01.

Previous studies showed that adipocyte-specific LRP1 inactivation in mice reduces diet-induced obesity and glucose intolerance but results in hyperlipidemia due to impaired clearance of triglyceride-rich lipoproteins (16). The adipocyte-specific LRP1 knockout mice are also protected from diet-induced lipid accumulation in the liver (16). In contrast, LRP1 inactivation in hepatocytes exacerbates HF diet-induced obesity, glucose intolerance, insulin resistance, and hepatosteatosis (11). Hepatic LRP1 inactivation also synergizes with dietary cholesterol to accelerate liver disease progression to steatohepatitis (12, 13). The current study revealed that the metabolic phenotype of LRP1 NPxY mutant mice is different from that observed in adipocyte- or hepatocyte-specific LRP1 knockout mice. When fed the LF chow diet, the LRP1 NPxY mutant mice displayed similar body weight and adiposity as C57BL/6J wild-type mice. In addition, both strains gained similar amounts of body weight and adiposity after feeding an HF diet (Fig. 1A, B). However, when fed the HFHC diet, the LRP1 NPxY mutant mice gained significantly less weight and adiposity than wild-type mice (Fig. 1A, B). The lower adiposity in HFHC diet-fed LRP1 NPxY mutant mice was reflected by their lower leptin expression levels in adipose tissues, leading to lower plasma leptin levels in HFHC diet-fed LRP1 NPxY mutant mice than in wild-type mice on the same diet or in mice fed the HF diet without cholesterol supplementation (Fig. 1C, D). However, adiponectin expression levels were comparable between wild-type and LRP1 NPxY mutant mice under all dietary conditions (Fig. 1E). The lower ratio of leptin to adiponectin expression observed in HFHC diet-fed LRP1 NPxY mutant mice suggests that their adipose tissues were less inflamed. Histological examination of the adipose tissues revealed similarly enlarged adipocytes and a comparable number of CLSs, indicative of macrophages surrounding dead adipocytes (23), in both wild-type and mutant mice fed the HF diet (Fig. 1F). In contrast, when the mice were fed HFHC diet, smaller adipocytes and fewer CLSs were observed in the LRP1 NPxY mutant mice than in wild-type mice (Fig. 1G). The reduced number of macrophages in the adipose tissues of HFHC diet-fed mutant mice was also evident by lower expression levels of EMR1 (F4/80), MCP1/CCL2, and MIP1α/CCL3 than that observed in wild-type mice, or in wild-type and mutant mice fed the HF diet (Fig. 1H). The reduced presence of macrophages and lower MCP1/CCL2 expression observed in HFHC diet-fed LRP1 NPxY mutant mice compared with those in wild-type mice suggested that the LRP1 NPxY mutant mice may be more resistant to HFHC diet-induced suppression of adipose tissue insulin sensitivity (24, 25). Indeed, the injection of insulin into HFHC diet-fed wild-type and LRP1 NPxY mutant mice followed by isolation of adipose tissues to assess insulin signaling revealed higher levels of AKT phosphorylation in the LRP1 NPxY mutant mice than that observed in wild-type mice (Fig. 1I).

### LRP1 distal NPxY motif mutation causes hepatic insulin resistance but does not exacerbate diet-induced hyperglycemia or hyperinsulinemia

**Fig. 2:**
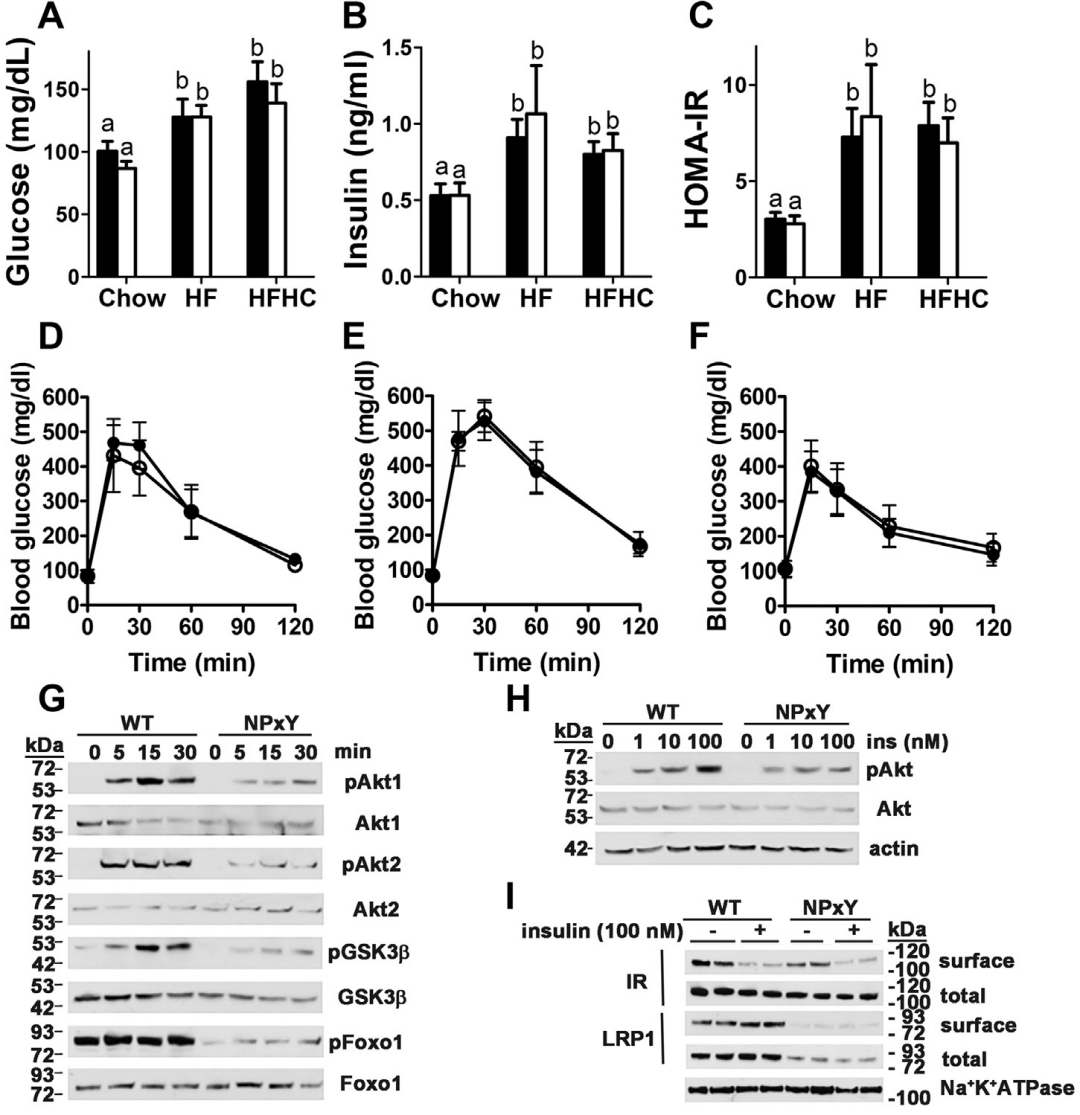
LRP1 distal NPxY motif mutation impairs insulin signaling in isolated hepatocytes but does not exacerbate diet-induced hyperglycemia and hyperinsulinemia. Plasma glucose (A) and insulin levels (B) of wild-type (WT, filled bars) and LRP1 NPxY mutant mice (open bars) after feeding a low-fat chow, HF, or HFHC diet for 16 weeks were determined after an overnight fast (5–15 per group). C: Homeostatic assessment of insulin resistance (HOMA-IR) index calculated from the data shown in panels (A) and (B). Differences between groups in each experiment were evaluated by one-way ANOVA with the Student-Newman-Keuls test for multiple group comparisons. Bars with different letters denote differences at *P* < 0.01. Glucose tolerance tests were performed by intraperitoneal injection of glucose (2 g/kg body weight) and measuring blood glucose levels over a 2-h period in wild-type (solid symbols) and LRP1 NPxY mutant (open symbols) mice after feeding chow (D), HF (E), or HFHC diets (F). G: Primary hepatocytes from WT and LRP1 NPxY mutant mice were stimulated with 100 nM insulin for the indicated time points. Phosphorylation and expression of AKT1, AKT2, GSK3β, and Foxo1 were analyzed by Western blot. H: Western blot analysis of dose-dependent phosphorylation of AKT in primary hepatocytes from WT and LRP1 NPxY mutant mice stimulated with insulin for 30 min. I: Western blot analysis of surface and total expression of insulin receptor and LRP1 in primary hepatocytes from WT and LRP1 NPxY mutant mice stimulated with 100 nM insulin for 5 min. All Western blots are representative images of three independent experiments.

Despite the reduced adiposity and adipose tissue inflammation, as well as higher insulin sensitivity in the adipose tissues of LRP1 NPxY mutant mice fed the HFHC diet, both wild-type and mutant mice showed similarly high fasting glucose and insulin levels, leading to a similar insulin resistance index regardless of the cholesterol content in the HF diet (Fig. 2A–C). Glucose tolerance tests performed in these animals confirmed no differences between wild-type and LRP1 NPxY mutant mice in response to glucose (Fig. 2D–F). These results are different than those observed in adipocyte-specific LRP1 knockout mice, which showed improved glucose tolerance and insulin sensitivity due to compensatory nutrient utilization for muscular thermogenesis (16), or liver-specific LRP1 knockout mice that showed exaggerated diet-induced hyperglycemia and hyperinsulinemia due to hepatic insulin resistance (11).

Additional experiments were performed with primary hepatocytes isolated from wild-type and LRP1 NPxY mutant mice to determine whether the LRP1 NPxY mutation affects hepatic insulin resistance. Results showed that the incubation of hepatocytes with insulin from 0 to 30 min resulted in increased phosphorylation of AKT1 and AKT2 in wild-type cells in a time- and concentration-dependent manner, but their phosphorylation levels were significantly attenuated in hepatocytes with LRP1 NPxY mutation (Fig. 2G, H). The LRP1 NPxY mutation also reduced insulin-induced phosphorylation of GSK3β and Foxo1 (Fig. 2G). The mechanism of impaired insulin signaling in the LRP1 NPxY mutant hepatocytes is due to reduced LRP1 level and its presence on the cell surface as shown previously (26). The addition of insulin, which typically stimulates LRP1 translocation to the cell surface (11), did not increase the number of the LRP1 NPxY mutant protein on the cell surface (Fig. 2I). Importantly, the NPxY motif mutation in LRP1 also reduced the number of insulin receptors on the cell surface (Fig. 2I), similar to that observed with hepatic LRP1 inactivation (11). Taken together, these data indicate that LRP1 NPxY mutation impairs hepatic insulin signaling. However, unlike the liver-specific LRP1 knockout mice, the impairment of hepatic insulin signaling did not further exacerbate diet-induced hyperglycemia, hyperinsulinemia, and insulin resistance.

### LRP1 distal NPxY motif mutation reduces dietary cholesterol-induced hypercholesterolemia

**Fig. 3:**
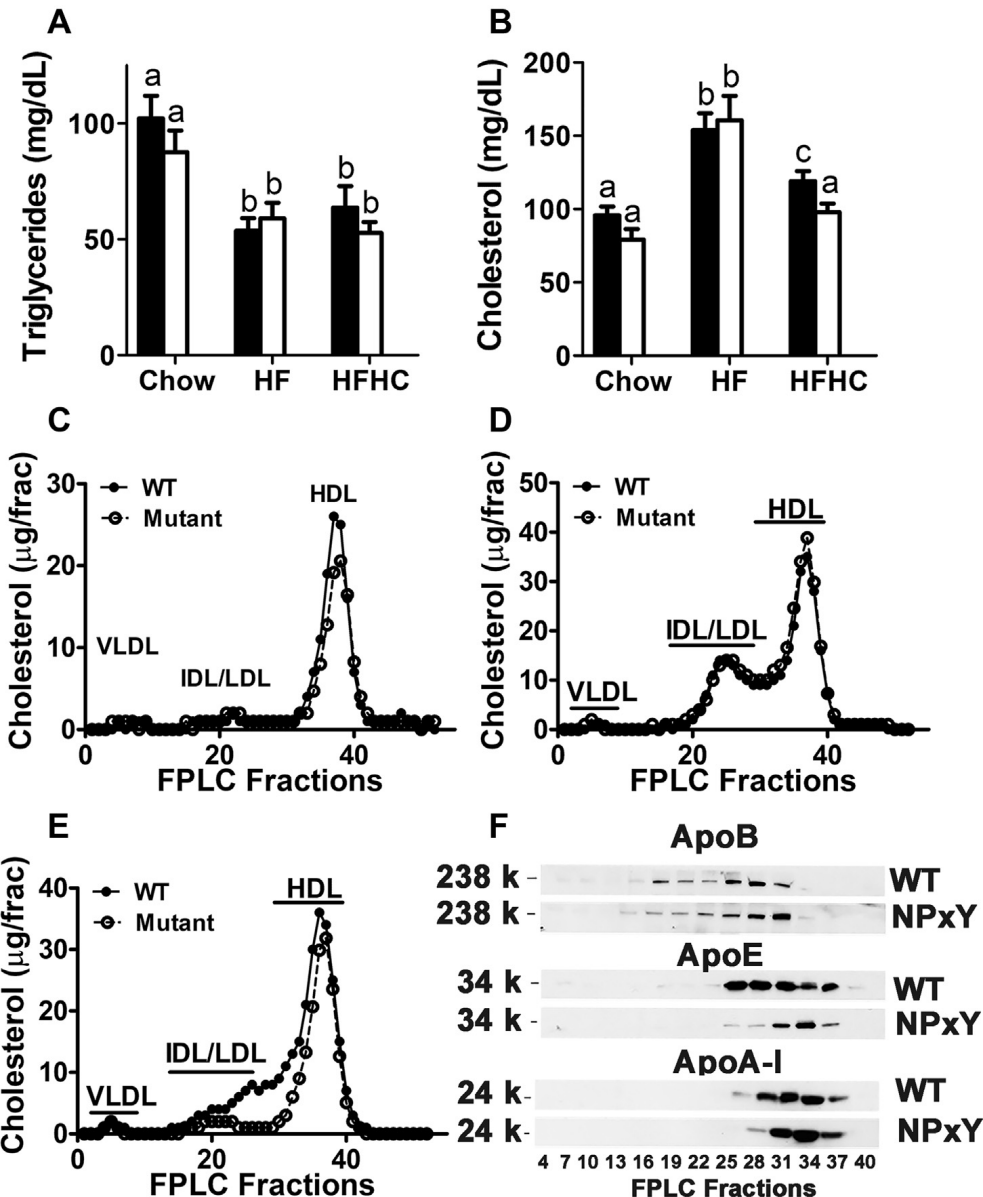
LRP1 distal NPxY motif mutation reduces dietary cholesterol-induced hypercholesterolemia. Plasma triglyceride (A) and cholesterol levels (B) levels of WT (filled bars) and LRP1 NPxY mutant mice (open bars) after feeding a low-fat chow, HF, or HFHC diet for 16 weeks were determined after an overnight fast (6–11 mice per group). Differences between groups in each experiment were evaluated by one-way ANOVA with the Student-Newman-Keuls test for multiple group comparisons. Bars with different letters denote differences at *P* < 0.01. C: Pooled plasma samples from fasting WT (filled symbols) and LRP1 NPxY mutant mice (open symbols) fed low-fat chow diet, HF diet (D) or HFHC diet (E) were fractionated by FPLC for lipoprotein analysis. F: Western Blot analysis of ApoB, ApoE, and ApoA-I distribution in plasma samples fractionated by FPLC from WT and LRP1 NPxY mutant mice after feeding an HFHC diet for 16 weeks.

The LRP1 NPxY mutation has been shown to improve hyperlipidemia in *ApoE*^*−/−*^ mice (26). To determine whether LRP1 NPxY mutation also improves hyperlipidemia due to chronic consumption of hypercaloric diet, which may partially alleviate insulin resistance caused by hepatic insulin signaling impairment, we compared plasma lipid levels between wild-type and the LRP1 NPxY mutant mice under all 3 dietary conditions. Results showed similar fasting plasma triglyceride levels in wild-type and mutant mice under all three dietary conditions (Fig. 3A). Chow-fed LRP1 NPxY mutant mice also showed a trend of lower plasma cholesterol levels than wild-type mice when the animals were fed the LF/low-cholesterol chow diet, but the difference did not reach statistical significance (Fig. 3B). Both groups displayed elevated plasma cholesterol levels when fed the HF diet without cholesterol supplementation (Fig. 3B). Interestingly, plasma cholesterol levels in the mutant mice were significantly lower than those observed in wild-type mice when fed the HFHC diet (Fig. 3B). Analysis of cholesterol distribution among the various lipoprotein classes revealed that chow-fed LRP1 NPxY mutant mice contained lower HDL-cholesterol levels (100 μg total in fractions 32–42) than HDL-cholesterol levels observed in chow-fed wild-type mice (122 μg total) (Fig. 3C), similar to that observed previously in liver-specific LRP1 knockout mice (10). Feeding the HF diet increased cholesterol levels of IDL/LDL fractions to a similar extent in both wild-type and mutant mice, but the difference in HDL levels was no longer evident (Fig. 3D). Surprisingly, the inclusion of additional cholesterol to the HF diet lowered cholesterol levels in the IDL/LDL fractions as well as fractions containing large HDL (Fig. 3E). Western blot analysis of apolipoproteins in each fraction confirmed that the identified IDL/LDL fractions contained mostly apoB-containing lipoproteins and the large HDL fractions contained mostly apoE-rich apoA-I-containing HDL (Fig. 3F). Thus, the lower plasma cholesterol levels in the HFHC diet-fed mutant mice than in HF diet-fed mice were due to lower levels of apoB-containing IDL/LDL (fractions 16–31) and apoA-I- and apoE-containing lipoproteins (fractions 25–31).

### LRP1 distal NPxY motif mutation suppresses dietary cholesterol-induced liver steatosis and inflammation

**Fig. 4:**
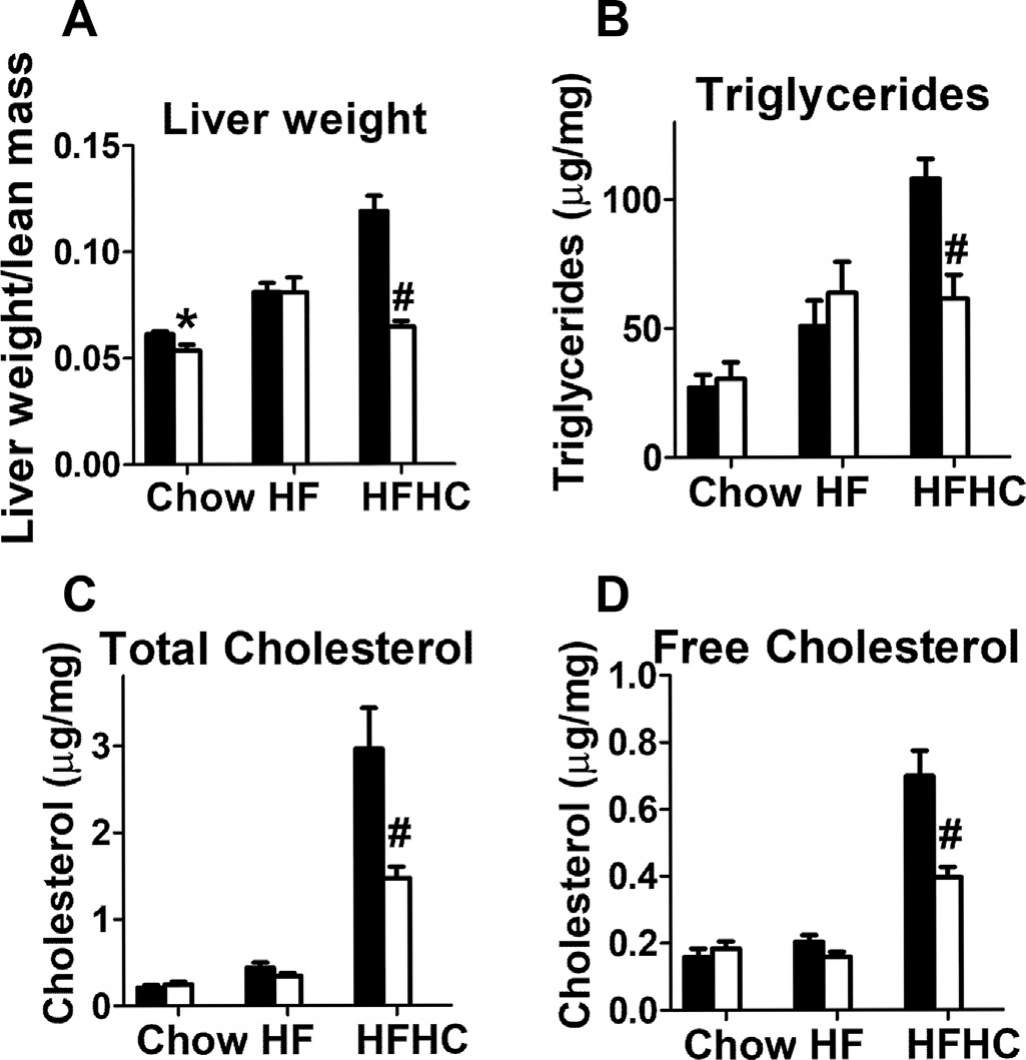
LRP1 distal NPxY motif mutation reduces dietary cholesterol-induced liver steatosis. A: Liver weight, (B) hepatic triglycerides, (C) hepatic total cholesterol and (D) hepatic free cholesterol of WT (filled bars) and LRP1 NPxY mutant mice (open bars) after feeding a low-fat chow, HF, or HFHC diet for 16 weeks (7–9 mice per group). Differences between groups in each experiment were evaluated by one-way ANOVA with the Student-Newman-Keuls test for multiple group comparisons. Bars with different letters denote differences at *P* < 0.01.

Previous studies have shown that hepatic LRP1 deficiency promotes HF diet-induced hepatic steatosis (11) and synergizes with dietary cholesterol to accelerate the progression of hepatosteatosis to steatohepatitis (13). In view of the data showing that the LRP1 NPxY mutant mice were resistant to HFHC diet-induced hypercholesterolemia, yet exhibited impaired hepatic insulin signaling, we compared liver phenotype in wild-type and mutant mice to explore whether the distal NPxY motif participates in LRP1 modulation of liver steatosis and inflammation. Results showed that both wild-type and LRP1 NPxY mutant mice displayed a similar increase in liver weight and accumulated similar amounts of triglycerides and cholesterol in the liver upon feeding the HF diet without cholesterol supplementation (Fig. 4A–D). Consistent with results reported in previous studies (13), cholesterol enrichment of the HF diet enhanced liver weight gain and accumulation of both triglycerides and cholesterol in wild-type mice (Fig. 4A–C). Interestingly and in contrast to results observed in wild-type and hepatocyte-specific LRP1 knockout mice (13), liver weight and steatosis were significantly lower in cholesterol-fed LRP1 NPxY mutant mice than in similarly fed control mice (Fig. 4A–D).

**Fig. 5:**
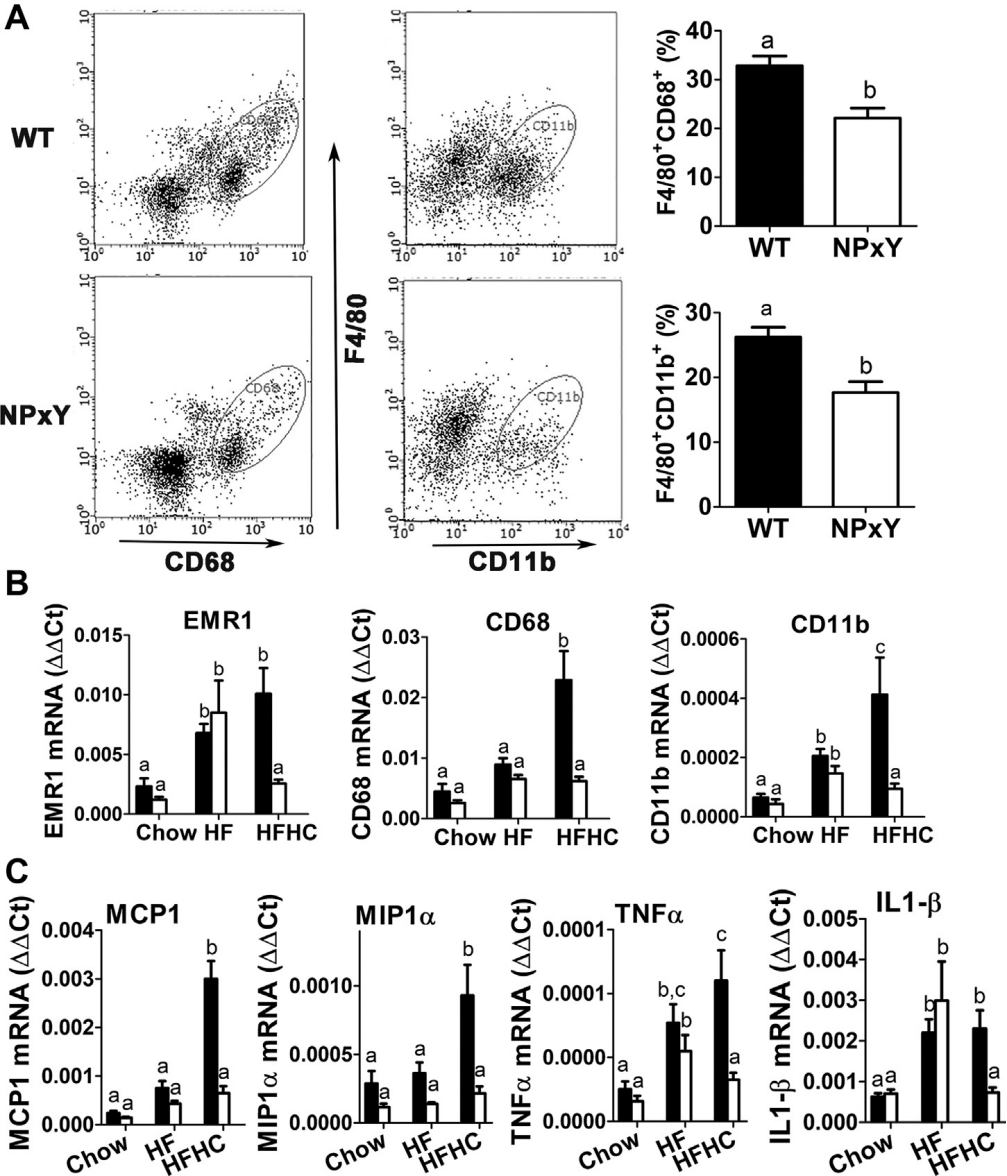
LRP1 distal NPxY motif mutation reduces dietary cholesterol-induced liver inflammation. A: The percentage of CD68^+^ cells (left panel) and CD11b^+^ cells (right panel) in livers of WT (filled bars) and LRP1 NPxY mutant mice (open bars) after feeding an HFHC diet for 16 weeks was quantified by flow cytometry (seven mice per genotype). B, C: Expression levels of EMR1, CD68, CD11b, MCP1, Mip1α, ΤΝFα, and IL-1β in livers of WT (filled bars) and LRP1 NPxY mutant mice (open bars) after feeding a low-fat chow, HF, or HFHC diet for 16 weeks. Data represent mean ± SEM from 7–10 mice per group. Differences between groups in each experiment were evaluated by one-way ANOVA with the Student-Newman-Keuls test for multiple group comparisons. Bars with different letters denote differences at *P* < 0.01.

Flow cytometry analysis of nonparenchymal cells in the livers of cholesterol-fed wild-type and LRP1 NPxY mutant mice revealed higher levels of both CD68^+^ and CD11b^+^ macrophages in the livers of wild-type mice than in the LRP1 NPxY mutant mice (Fig. 5A). Analysis of gene expression profile in the livers of wild-type and LRP1 NPxY mutant mice showed higher levels of EMR1 (F4/80) mRNA in both wild-type and LRP1 NPxY mutant mice after HF feeding, but the mRNA levels of CD68 and CD11b in these animals were similar to that observed in chow-fed mice (Fig. 5B). These data suggest that under the current experimental conditions, HF feeding leads predominantly to the activation of CD11b^*−*/*−*^ Kupffer cells instead of recruitment of CD68^+^ cells into the liver (Fig. 5B). Interestingly, expression of EMR1, CD68, and CD11b was further enhanced in wild-type mice upon feeding the HFHC diet, indicative of both Kupffer cell activation and circulating monocytes/macrophage recruitment. In contrast, the inclusion of cholesterol in the HF diet reduced the expression of these macrophage markers in the livers of LRP1 NPxY mutant mice to levels observed when the mice were fed the LF chow diet (Fig. 5B). The differences in the number of macrophage-like cells in the livers of wild-type and LRP1 NPxY mutant mice were likely due to differences in expression of MCP1 (CCL2) and MIP1α (CCL3) that are responsible for monocyte recruitment (Fig. 5C). As a result, expression levels of inflammatory cytokines such as TNFα and IL-1β were also found to be elevated in both wild-type and mutant mice under HF feeding conditions, and the expression levels of these cytokines were further enhanced in wild-type mice but reduced in the LRP1 mutant mice when fed the HFHC diet (Fig. 5C). Taken together, these results indicated that distal NPxY mutation in LRP1 has no effect on HF diet-induced hepatosteatosis but reduces HFHC acceleration of fatty liver disease to the inflammatory stage.

### LRP1 NPxY mutation improves plasma lipid clearance to suppress cholesterol diet-induced hypercholesterolemia

**Fig. 6:**
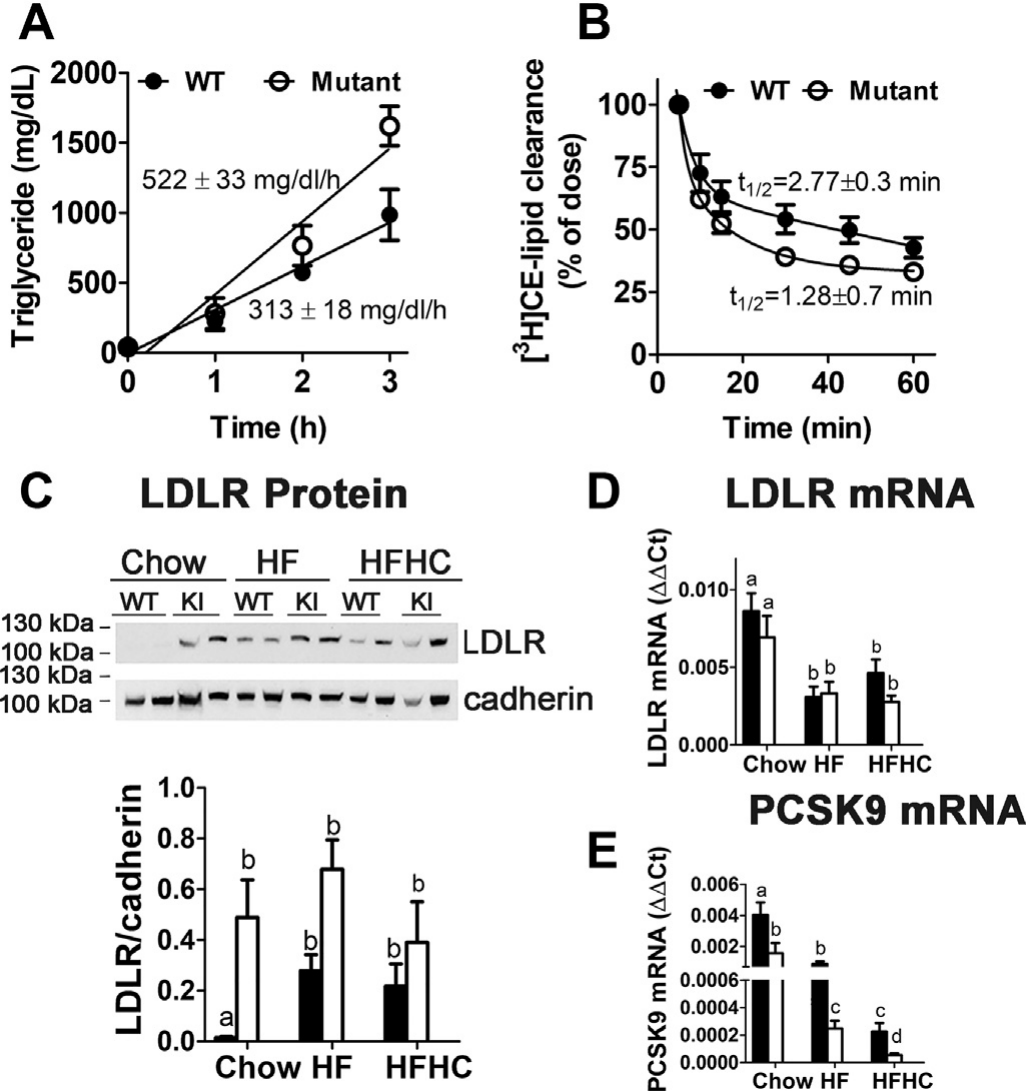
LRP1 distal NPxY motif mutation does not affect VLDL synthesis but improves plasma lipid clearance via elevated LDL receptor (LDLR) levels in the liver. A: VLDL synthesis/secretion and (B) plasma lipid clearance in WT (filled symbols) and LRP1 NPxY mutant mice (open symbols) fed HFHC diet for 16 weeks (5–9 mice per group). C: Representative Western blot analysis of hepatic LDLR and cadherin levels in WT and LRP1 NPxY knock-in mutant mice (KI) fed chow, HF, or HFHC diets. LDLR levels from four mice in each group were presented in the bar graph as mean ± SEM. Bars with different letters denote differences at *P* < 0.01. The mRNA levels of LDLR and PCSK9 in the livers of wild-type (filled bars) and LRP1 NPxY mutant mice (filled bars) after feeding chow, HF, or HFHC diets are shown in panels D and E, respectively. Differences in mRNA expression levels were evaluated by one-way ANOVA with the Student-Newman-Keuls test for multiple group comparisons. Bars with different letters denote differences at *P* < 0.01.

The mechanism by which mutation of the distal NPxY motif in LRP1 alleviates dietary cholesterol-induced hypercholesterolemia was explored by comparing VLDL synthesis/secretion and plasma lipid clearance between wild-type and LRP1 NPxY mutant mice after feeding the HFHC diet for 16 weeks. Results showed that the lower plasma cholesterol levels in LRP1 NPxY mutant mice were not due to reduced VLDL synthesis and secretion. In fact, rate of VLDL secretion, as monitored by changes in plasma triglyceride levels after Poloxamer 407 injection to block lipolysis, was significantly higher (*P* < 0.01) in LRP1 NPxY mutant mice than in wild-type mice (Fig. 6A). In contrast, when lipid emulsions containing [^3^H]cholesteryl oleoyl ether were injected intravenously into the mice, the initial fast rate of plasma clearance of these artificial lipoproteins was found to be ∼ twofold higher in LRP1 NPxY mutant mice than in wild-type mice (Fig. 6B, *P* < 0.01).

To identify the mechanism responsible for the difference in lipoprotein clearance rates, we examined LDL receptor levels in the livers of wild-type and LRP1 NPxY mutant mice. Consistent with previous reports that LRP1 NPxY mutation leads to increased LDL receptor expression, we found higher LDL receptor levels in the mutant mice under all three dietary conditions (Fig. 6C). Feeding of the HF diet resulted in increased LDL receptor levels in both wild-type and LRP1 NPxY mutant mice, and the increase was less pronounced when the mice were fed the HFHC diet (Fig. 6C). Thus, the higher rate of plasma lipoprotein clearance observed in LRP1 NPxY mutant mice than in wild-type mice is due to the increased LDL receptor levels in the mutant mice. The differences in LDL receptor levels were not due to differences in LDL receptor gene expression since higher LDL receptor mRNA levels were observed in both wild-type and LRP1 NPxY mutant mice under chow-fed conditions than those observed in animals fed HF or HFHC diets (Fig. 6D). Noting that the LDL receptor level is also regulated at the post-translational level through PCSK9-enhanced degradation, we measured PCSK9 mRNA levels in these animals and found that the LRP1 NPxY mutant mice displayed lower PCSK9 mRNA level than the wild-type mice under all three dietary conditions (Fig. 6E). These results indicate that the lower plasma cholesterol levels in HFHC diet-fed LRP1 NPxY mutant mice than in wild-type mice are due to increased LDL receptor-mediated plasma lipoprotein clearance as a consequence of reduced PCSK9-mediated LDL receptor degradation.

### LRP1 NPxY mutation alleviates LXR activation in the liver

**Fig. 7:**
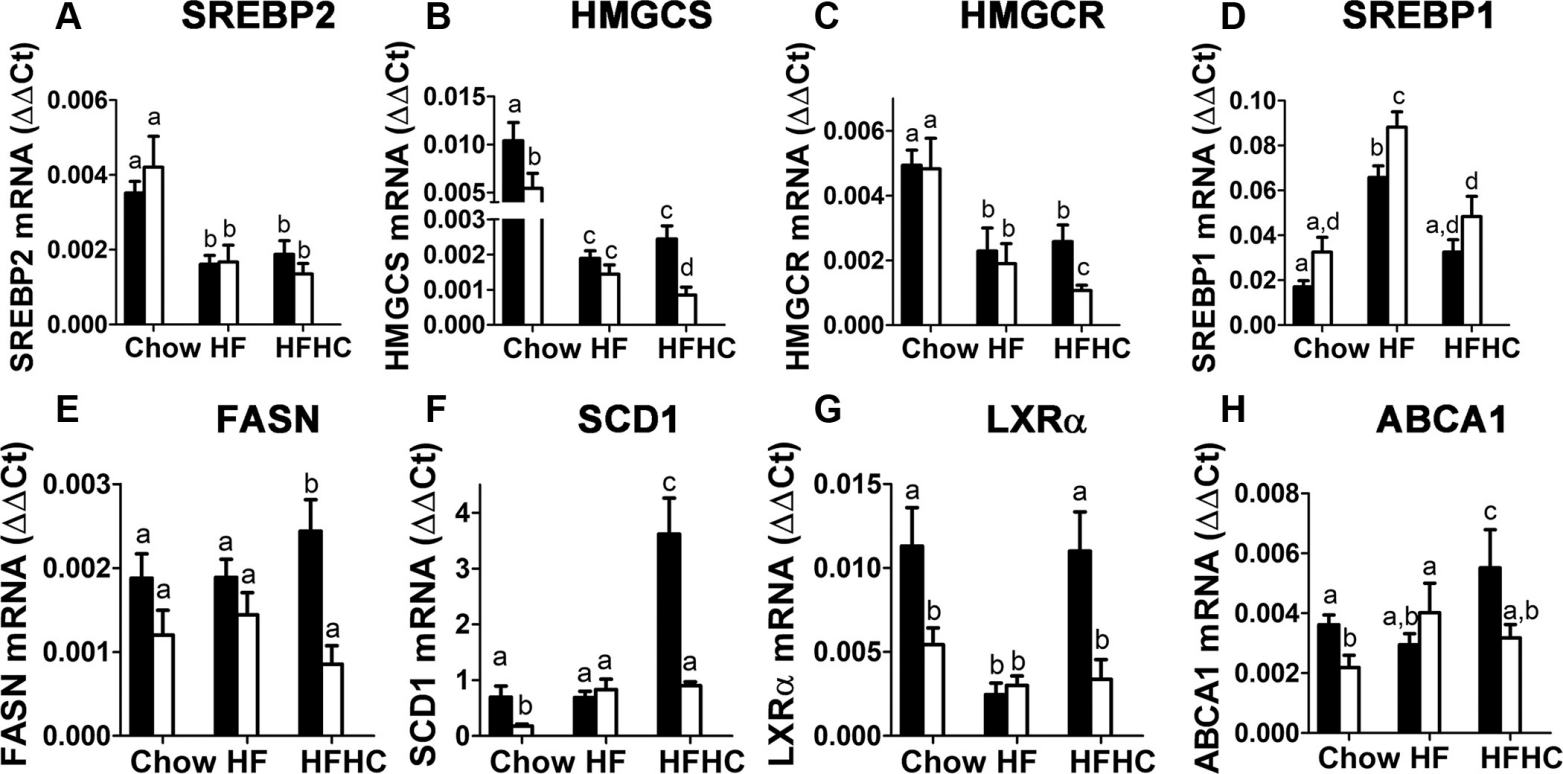
LRP1 distal NPxY motif mutation alleviates cholesterol-induced LXR activation in the liver. Expression levels of (A) SREBP2, (B) HMGCS, (C) HMGCR, (D) SREBP1, (E) FASN, (F) SCD1, (G) LXRα, and (H) ABCA1 in livers of WT (filled bars) and LRP1 NPxY mutant mice (open bars) after feeding a low-fat chow, HF, or HFHC diet for 16 weeks. Data represent mean ± SEM from 7–10 mice per group. Differences between groups in each experiment were evaluated by one-way ANOVA with the Student-Newman-Keuls test for multiple group comparisons. Bars with different letters denote differences at *P* < 0.01.

The mechanism by which mutation of the distal NPxY motif in LRP1 reduces PCSK9 expression to alleviate dietary cholesterol-induced hypercholesterolemia and steatohepatitis was investigated by comparing the expression of lipid metabolism genes in the liver of wild-type and LRP1 NPxY mutant mice under various dietary conditions. Results showed that HF diet, both with or without additional cholesterol supplementation, reduced expression of SREBP2 and SREBP2-sensitive genes such as HMG CoA synthase (HMGCS) and HMG CoA reductase (HMGCR) in addition to the reduction of the LDL receptor (LDLR). However, no significant differences were observed between wild-type and LRP1 NPxY mutant mice when these animals were fed the HF diet without cholesterol supplementation (Fig. 7A–C). The addition of cholesterol to the HF diet led to further reduction of HMGCS and HMGCR expression in LRP1 NPxY mutant mice but not in wild-type mice (Fig. 7B, C), likely reflecting the lower cholesterol levels in the livers of HFHC diet-fed LRP1 NPxY mutant mice. HF feeding also led to increased expression of SREBP1 in the livers (Fig. 7D). Interestingly, the increase was more pronounced in LRP1 NPxY mutant mice than in wild-type mice (Fig. 7D). The addition of cholesterol to the HF diet lowered SREBP1 expression levels, and differences were no longer observed between wild-type and LRP1 NPxY mutant mice (Fig. 7D). Despite differences in SREBP1 expression between chow- and HF diet-fed animals, mRNA levels of fatty acid synthase (FASN) and stearoyl-CoA desaturase-1 (SCD1) were found to be similar between chow- and HF diet-fed animals (Fig. 7E, F). Furthermore, cholesterol supplementation to the HF diet resulted in increased expression of FASN and SCD1 in wild-type but not in LRP1 NPxY mutant mice (Fig. 7E, F).

Since the expression of FASN, SCD1, and PCSK9 may also be regulated by the transcription factor LXR (27, 28, 29, 30), we also measured LXRα mRNA levels in the animals and found that hepatic expression of this transcription factor was reduced in LRP1 NPxY mutant mice compared with wild-type mice (Fig. 7G). Moreover, the dietary cholesterol-induced LXRα expression observed in wild-type mice was not evident in HFHC diet-fed LRP1 NPxY mutant mice. Additionally, expression of another LXR-responsive gene, ABCA1, was also lower in the LRP1 NPxY mutant mice fed chow or HFHC diet (Fig. 7). Taken together, these results suggest that NPxY motif mutation in LRP1 abrogates cholesterol-induced LXR activation, thereby decreasing PCSK9 expression to suppress cholesterol-induced hypercholesterolemia. The reduction in ABCA1 expression is also consistent with lower plasma HDL levels observed in the LRP1 NPxY mutant mice (Fig. 3B), whereas the lower expression levels of the lipogenic genes may account for the reduced steatosis and lower inflammation in the livers of HFHC diet-fed LRP1 NPxY mutant mice.

### LRP1 NPxY mutation reduces cholesterol-induced systemic and neuroinflammation

**Fig. 8:**
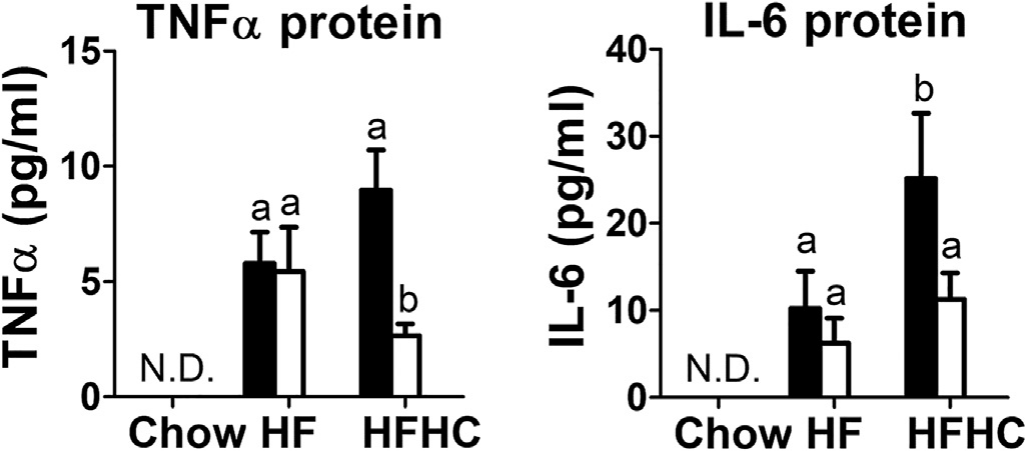
LRP1 distal NPxY motif mutation reduces dietary cholesterol-induced plasma cytokine levels. Plasma levels of TNFα and IL-6 were measured in wild-type (filled bars) and LRP1 NPxY mutant (open bars) mice after feeding chow-, HF-, or HFHC diets. Data represent mean ± SEM from six mice in each group. Differences between groups in each experiment were evaluated by one-way ANOVA with the Student-Newman-Keuls test for multiple group comparisons. Bars with different letters denote differences at *P* < 0.01. N.D., not detectable.

The reduced expression of inflammatory cytokine genes in the adipose tissues and liver of HFHC diet-fed LRP1 NPxY mutant mice compared with similarly fed wild-type mice suggests that global NPxY mutation in LRP1 may protect against cholesterol-induced systemic inflammation. To test this possibility, cytokine levels in the plasma of wild-type and LRP1 NPxY mutant mice were compared under all three dietary conditions. Although plasma IL-1β levels were below the detection limit under all conditions tested, we found that plasma TNFα and IL-6 levels were significantly higher in HF diet-fed mice than in chow-fed animals, but no difference was observed between wild-type and LRP1 NPxY mutant mice (Fig. 8). Plasma IL-6 levels were further increased in HFHC diet-fed wild-type mice, and this increase was not observed in HFHC diet-fed LRP1 NPxY mutant mice (Fig. 8). These data indiated that the NPxY mutation in LRP1 specifically blunted cholesterol exacerbation of systemic inflammation.

**Fig. 9:**
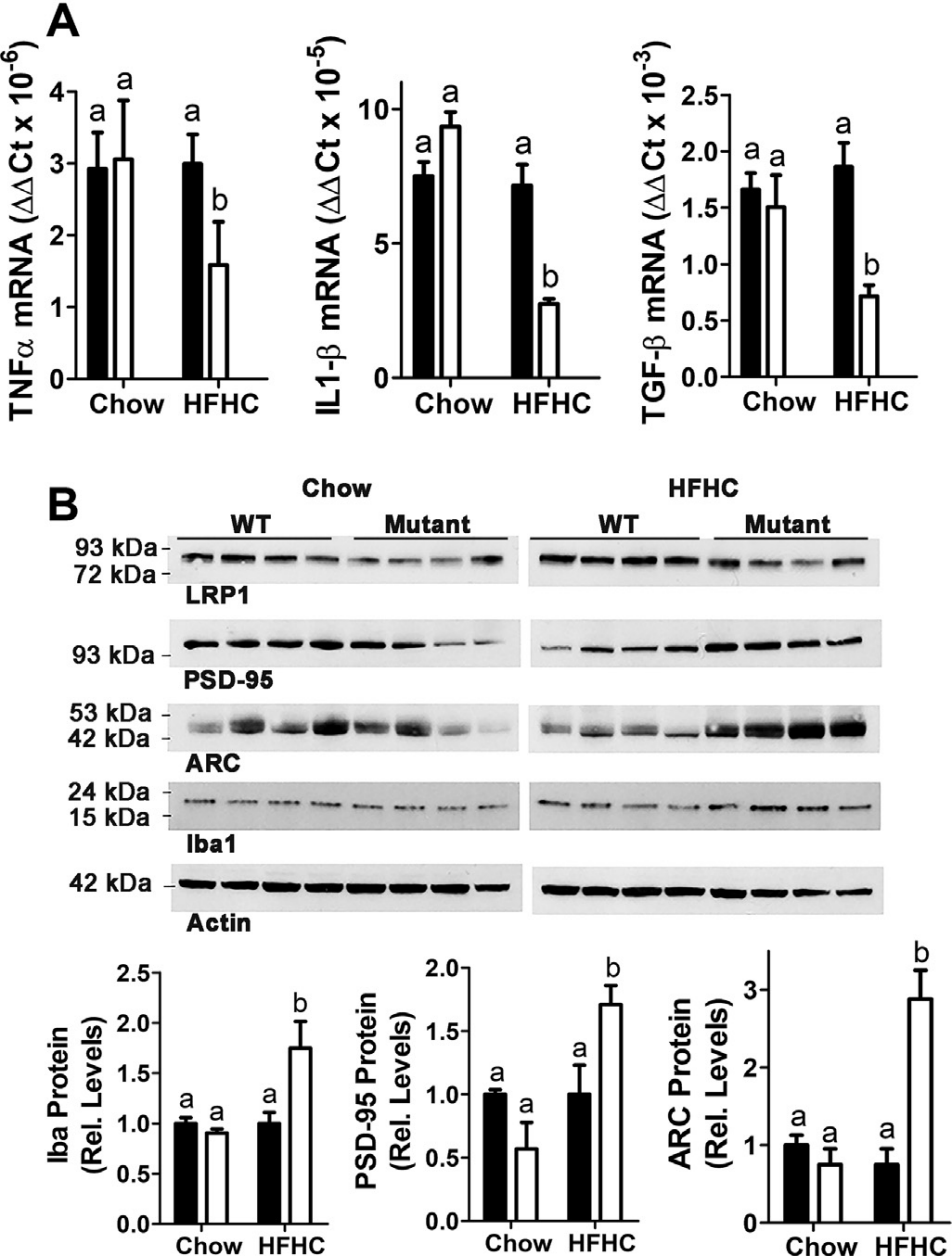
LRP1 distal NPxY motif mutation reduces cholesterol-induced neuroinflammation. A: Expression levels of TNFα, IL1β, and TGFβ mRΝΑ. B: Western blot analysis of LRP1, PSD-95, ARC, and Iba1 proteins in brains of WT (filled bars) and LRP1 NPxY mutant mice (open bars) after feeding a low-fat chow or HFHC diet for 16 weeks. Data represent mean ± SEM from 7–8 mice per group. Differences between groups in each experiment were evaluated by one-way ANOVA with the Student-Newman-Keuls test for multiple group comparisons. Bars with different letters denote differences at *P* < 0.01.

A diet high in fat and cholesterol content has also been shown to increase neuroinflammation, leading to cognitive dysfunction in mice similar to Alzheimer's disease characteristics in humans (31). Hence, we also examined how LRP1 NPxY mutation may impact brain inflammation in response to HFHC feeding. Analysis of mRNA in the brain of chow- and HFHC diet-fed wild-type and LRP1 NPxY mutant mice revealed a significant reduction in the expression of proinflammatory cytokines such as TNFα, IL1-β, and transforming growth factor beta in HFHC diet-fed LRP1 NPxY mutant mice compared with wild-type mice (Fig. 9A). Interestingly, Western blot analysis revealed higher levels of ionized calcium-binding protein molecule-1 (Iba1) in LRP1 NPxY mutant mice, indicative of microglia activation (Fig. 9B). However, no difference in the levels of amyloid β protein, β-site APP cleaving enzyme 1, and (phospho)tau proteins was observed in Western blot analysis of brain lysates prepared from chow- or HFHC diet-fed wild-type and LRP1 NPxY mutant mice (data not shown). Nevertheless, the levels of the synaptic strength regulator PSD-95 and ARC, a protein that maintains synaptic potentiation, were found to be higher in HFHC diet-fed LRP1 NPxY mutant mice than in wild-type mice (Fig. 9B). Previous studies have shown that ARC expression is decreased on HFHC diet (32). Therefore, the differences in ARC levels may reflect differences in brain cholesterol in wild-type and LRP1 NPxY mutant mice. Taken together, these data indicate that LRP1 NPxY mutation limits HFHC diet-induced neuroinflammation and preserves cognition.

## Discussion

Previous studies have shown that mutation in the LRP1 distal NPxY motif does not affect ligand binding or its regulation of cell signaling events but compromises the rate of LRP1 endocytosis and recycling that leads to delayed postprandial lipid clearance, accelerated atherosclerosis, and increased inflammatory response in *Ldlr*^*−/−*^ mice (33, 34). In contrast, LRP1 distal NPxY mutation was shown to improve dyslipidemia and atherosclerosis in *ApoE*^*−/−*^ mice (26). These controversies highlight the importance of exploring the influence of this mutation in wild-type mice with physiologically regulated expression of apoE and LDL receptors. This study showed no differences between wild-type and LRP1 NPxY mutant mice when fed either the LF chow diet or an HF diet without cholesterol supplementation. Both groups responded similarly to HF feeding with hyperglycemia, hyperinsulinemia, and hyperlipidemia. Both wild-type and LRP1 NPxY mutant mice also showed increased adiposity with robust adipose tissue inflammation and liver steatosis, but neither group showed extensive liver inflammation when fed the HF diet without cholesterol supplementation. In contrast, when the animals were fed an HFHC diet, the LRP1 NPxY mutation prevents hypercholesterolemia, reduces adipose and brain tissue inflammation, and limits steatotic liver progression to steatohepatitis. Nevertheless, insulin signaling is impaired in LRP1 NPxY mutant hepatocytes and this mutation does not exacerbate or protect against HFHC diet-induced hyperglycemia, hyperinsulinemia, and insulin resistance. Thus, mutation and dysfunction of the distal NPxY motif in LRP1 is not sufficient to explain the relationship between *LRP1* polymorphism and insulin resistance in patients with metabolic syndrome (5).

The metabolic phenotype of the LRP1 NPxY mutant mice is different from that observed in *aLrp1*^*−/−*^ mice with adipocyte-specific LRP1 inactivation. The *aLrp1*^*−/−*^ mice are resistant to diet-induced obesity and are insulin sensitive with improved glucose tolerance compared with wild-type mice (16). The metabolic benefits of adipocyte LRP1 inactivation are due to impaired adipocyte differentiation (14, 15), leading to the redistribution of lipid nutrients to muscle cells to support thermogenesis (16). This study showed that the LRP1 NPxY mutant mice are susceptible to HF diet-induced adiposity and leptin expression, thereby indicating that the distal NPxY mutation in LRP1 influences neither adipocyte differentiation nor nutrient uptake for expansion of the adipocytes. The smaller adipocytes observed in HFHC diet-fed LRP1 NPxY mutant mice are likely due to the redistribution of nutrients to the liver as a consequence of the increased hepatic LDL receptor level in these animals, with less nutrients transported to adipocytes for storage. These results are consistent with in vitro data showing that the extracellular domain collaborates with the proximal NPxY motif in the cytoplasmic domain of LRP1, but the distal NPxY motif is not necessary, to modulate intracellular lipid accumulation (35). Hence, the LRP1 NPxY mutant mice are similar to wild-type mice in HF diet-induced hypercholesterolemia, hyperglycemia, insulin resistance, and hepatosteatosis.

The metabolic phenotype of LRP1 NPxY mutant mice is also different from that observed in *hLrp1*^*−/−*^ mice with hepatocyte-specific LRP1 inactivation. In particular, the LRP1 NPxY mutant mice are similar to wild-type mice in response to HF feeding, while HF diet-fed *hLrp1*^*−/−*^ mice are predisposed to metabolic disease with more robust insulin resistance, dyslipidemia, and hepatosteatosis compared with HF diet-fed wild-type mice (11). In addition, hepatic LRP1 deficiency synergizes with dietary cholesterol to exaggerate HF diet-induced liver diseases by accelerating hepatosteatosis progression to steatohepatitis (13), whereas distal NPxY mutation in LRP1 was found to limit obesity, prevent dyslipidemia, and suppress fatty liver disease progression in response to HFHC diet. However, despite these improvements, the LRP1 NPxY mutant mice are equally susceptible to HFHC diet-induced hyperglycemia, hyperinsulinemia, and insulin resistance. The selective metabolic improvement observed in HFHC diet-fed LRP1 distal NPxY mutant mice is likely due to the elevated rate of plasma lipoprotein clearance in HFHC diet-fed LRP1 NPxY mutant mice and the lower hepatic cholesterol levels than in HFHC diet-fed wild-type mice.

The higher rate of plasma lipoprotein clearance observed in HFHC diet-fed LRP1 NPxY mutant mice is likely due to higher hepatic LDL receptor levels than those in HFHC diet-fed wild-type mice, similar to that observed in *ApoE*^*−/−*^ mice (26). In the current study, we found that the difference in the hepatic LDL receptor level between wild-type and LRP1 NPxY mutant mice is not due to transcriptional difference in the LDL receptor expression but rather the lower expression level of PCSK9, a protein that induces internalization and degradation of the LDL receptor (36). The lower PCSK9 expression level in HFHC diet-fed LRP1 NPxY mutant mice is not due to differences in SREBP2 level or activity but is due to lower LXRα expression. The reduced LXRα level and activity also accounted for the reduced expression of ABCA1 and other LXR-responsive lipogenic genes, thereby resulting in lower plasma HDL levels and hepatosteatosis observed in LRP1 NPxY mutant mice. The importance of the LRP1 distal NPxY motif for LXR activation has been demonstrated in macrophages previously (34).

The improved liver phenotype of LRP1 NPxY mutant mice mimics the phenotype of *macLrp1*^*−/−*^ mice with macrophage-specific LRP1 inactivation (37). When bred to *Ldlr*^*−/−*^ background, the *Ldlr*^*−/−*^*macLrp1*^*−/−*^ mice were shown to display less hepatic inflammation than *Ldlr*^*−/−*^ mice when fed a Western-type diet (37). However, the *Ldlr*^*−/−*^*macLrp1*^*−/−*^ mice also gained less weight and had improved glucose tolerance compared with *Ldlr*^*−/−*^ mice (37). Thus, the metabolic phenotype of LRP1 NPxY mutant mice cannot be attributed to LRP1 dysfunction in macrophages. Interestingly, in another model of LRP1 NPxY mutant mice, in which the tyrosine residue in distal NPxY motif was mutated to phenylalanine, the LRP1 NPxF mutation was found to increase macrophage cholesterol accumulation and promote inflammation to accelerate atherosclerosis (34). The difference between the LRP1 NPxF mutant mouse study and our current results is that the LRP1 NPxF mutant mouse study was conducted after mating with *Ldlr*^*−/−*^ mice where Western diet feeding led to excess cholesterol accumulation in macrophages, whereas our study was conducted in wild-type background with physiologically regulated expression of the LDL receptor. We found that hepatic LDL receptor elevation due to impaired LXR-mediated PCSK9 response lowers plasma cholesterol levels in HFHC diet-fed LRP1 NPxY mutant mice. Thus, we posit that hepatic LDL receptor-mediated lipoprotein uptake and degradation pathway is responsible for the protection against HFHC diet-induced liver disease in LRP1 NPxY mutant mice.

The LRP1 NPxY mutant mice have also been shown to be defective in clearance of β-amyloid peptides (38). Interestingly, despite this defect, less extracellular β-amyloid peptides and reduced plaque deposition were found in Alzheimer's disease mice that also carry the LRP1 distal NPxY mutation (38). The current study revealed that the LRP1 NPxY mutation also reduces HFHC diet-induced brain inflammation. In view of studies showing a direct link between HFHC diet, hypercholesterolemia, and β-amyloid accumulation in mice (31), the improved brain phenotype observed in HFHC diet-fed LRP1 mutant mice compared with wild-type mice may also be related to the elevated rate of plasma lipoprotein clearance and the suppression of diet-induced hypercholesterolemia observed in these animals. Previous studies have shown the LRP1 NPxY mutation increases surface expression of the NR2B subunit of the N-methyl-D-aspartate receptor in the brain, resulting in hyperactivity and changes in spatial and reversal learning (39). Hypercholesterolemia in an Alzheimer's disease mouse model has also been shown to promote hyperactivity with spatial learning impairment (40). How does the LRP1 distal NPxY mutation cooperate with HFHC diet to influence activity and learning behavior remains to be determined.

In summary, this study showed that global dysfunction of LRP1 due to distal NPxY motif mutation is protective against HFHC diet-induced dyslipidemia, fatty liver disease, and neuroinflammation. This protective effect is not observed with HF diet without cholesterol supplementation. The mechanism is likely related to the increase in LDL receptor levels in the liver, resulting in increased plasma clearance of atherogenic lipoproteins. This phenotype is different from that observed with LRP1 inactivation in specific tissues, thus highlighting the importance of an integrative approach to evaluate how protein mutation and/or dysfunction at the organismal level may impact physiology.

### Data availability

The data supporting this study are available in the article and are available from the corresponding author upon request.

## Conflict of interest

The authors declare that they have no conflicts of interest with the contents of this article.
