# Supplementary material for: Mutation in the distal NPxY motif of LRP1 alleviates dietary cholesterol-induced dyslipidemia and tissue inflammation
Source: J Lipid Res. 2020 Dec 15;62:100012. doi: 10.1194/jlr.RA120001141 (PMC7859857; doi:10.1194/jlr.RA120001141)
Supplement: Supplemental Tables S1 to S3 [file mmc1.pdf]

Supplemental Table S1. Macronutrient composition of diets

**Macronutrients**	Chow (Teklad LM-485)		HF (Research Diets D12331)		HFHC (Research Diets D12108C)	
	% by weight	% kcal	% by weight	% kcal	% by weight	% kcal
Protein	19.1	25	23.0	16.4	23.0	20.0
Fat	5.8	17	35.8	58.0	20.0	40.0
(cholesterol)					(1.25)	
Carbohydrate	44.3	58	35.5	25.5	45.0	40.0
(sucrose)	(n.d.)		(17.5)	(12.5)	(12.6)	(11.1)

n.d. = not determined

Supplemental Table S2. Primer sequences used for RT-PCR amplification of RNA

**Gene**	**Forward Primer**	**Reverse Primer**
EMR1	TGTCTGACAATTGGGATCTGCCCT	ATACGTTCCGAGAGTGTTGTGGCA
CD68	TTTCTCCAGCTGTTCACCTTGA	CCCGAAGTGTCCCTTGTCA
CD11b	GCTCAGAGGTTCTTCACAGCTATG	CAAGCCCATGGCACTCATG
MCP1/CCL2	CCTCCTCCACCACCATGCA	CCAGCCGGCAACTGTGA
MIP1α	TTTGAAACCAGCAGCCTTTGCTCC	TCAGGCATTCAGTTCCAGGTCAGT
TNFα	ATCCGCGACGTGGAACTG	ACCGCCTGGAGTTCTGGAA
IL1-β	CTACAGGCTCCGAGATGAACAAC	TCCATTGAGGTGGAGAGCTTTC
SREBP2	TGCCAGCAGATGATTGTCAAG	AGAGACAAAAAGGTGGAAAGAAGTG
HMGCS	GACAAGAAGCCTGCTGCCATA	CGGCTTCACAAACCACAGTCT
HMGCR	ATGCCTTGTGATTGGAGTTGGCAC	GCCGGGAAGAATGTCATGAACAC
SREBP1	CGGCCCCTTCCCTCTACTC	GATACCACGATTGTTTGGAAGTG
LDLR	TCTGATGCGTCGCTGGGTCATC	CCGTCTCTACACTGGAACTCGTTC
PCSK9	CCAGAGGCTACAGATTGAACAAAC	TCACTGCTCATCTTCACCAAGAA
ABCA1	ACCCACCCTACGAACAACATGAGT	AAAGTTTCCAACAACACCGGGAGC
LXRα	CGACAGAGCTTCGTCCACAA	GCTCGTTCCCCAGCATTTT
FASN	CAAGCAGAATTTGTCCACCTTTAA	TCTCTAGAGGGCTTGCACCAA
SCD1	ATAACCGAATTCATGCCGGCCCAC ATGCT	GCTCAACTGCAGTCAGCTACTCTTGT GACTCC
Cyclophilin	TCATGTGCCAGGGTGGTGAC	CCATTCAGTCTTGGCAGTGC

Supplemental Table S3. Antibodies used for Western blot analysis

Antibodies	Supplier	Identifier
AKT1	Cell signaling	2967
AKT2	Cell signaling	5239
Phospho-AKT	Cell signaling	4056
β-actin	Cell signaling	4967
GSK3β	Cell signaling	9315
Phospho-GSK3β	Cell signaling	9336
Foxo1	Cell signaling	2880
Phospho-Foxo1	Cell signaling	9461
Insulin receptor	Cell signaling	3025
LRP1	Abcam	ab92544
Na^+K^+ATPase	Hybridoma bank	a6f
LDLR	Abcam	ab30532
PSD-95	Cell Signaling	3409
Iba1	Abcam	ab178846
Arc	Santa Cruz	sc-17839
apoB	Abcam	ab20737
apoE	Santa Cruz	sc-6384
apoA1	Abcam	ab7614
CD45 APC-eFluor 780	ebioscience	47-0451-82
CD68 PE-Cy7	ebioscience	25-0681-80
CD11b APC	ebioscience	17-0112-82
F4/80 BB700	BDBiosciences	746070
F4/80	Abcam	Ab6640
